# Caveolin-1 as a promoter of tumour spreading: when, how, where and why

**DOI:** 10.1111/jcmm.12030

**Published:** 2013-03-23

**Authors:** Rebecca Senetta, Giulia Stella, Ernesto Pozzi, Niccolo Sturli, Daniela Massi, Paola Cassoni

**Affiliations:** a Department of Medical Sciences, University of TurinTurin, Italy; b Department of Molecular Medicine, Section of Pneumology, Laboratory of Biochemistry & Genetics, University and Fondazione IRCCS Policlinico San MatteoPavia, Italy; c Policlinico di Monza and Department of Molecular Medicine, University of PaviaMonza, Italy; d Department of Critical Care Medicine and Surgery, University of FlorenceFlorence, Italy

**Keywords:** caveolin-1, cancer, metastases, biomarkers, targeted therapy

## Abstract

Caveolae are non-clathrin invaginations of the plasma membrane in most cell types; they are involved in signalling functions and molecule trafficking, thus modulating several biological functions, including cell growth, apoptosis and angiogenesis. The major structural protein in caveolae is caveolin-1, which is known to act as a key regulator in cancer onset and progression through its role as a tumour suppressor. Caveolin-1 can also promote cell proliferation, survival and metastasis as well as chemo- and radioresistance. Here, we discuss recent findings and novel concepts that support a role for caveolin-1 in cancer development and its distant spreading. We also address the potential application of caveolin-1 in tumour therapy and diagnosis.

- Introduction
- WHEN: Cav1 expression and disease stage
- HOW: Cav1 may promote tumour spreading
  - Cav1 and cell-cycle modulation
  - Cav1 and growth factors signalling
  - Cav1 and Rho-GTPases
  - Cav1 and integrins
  - Cav1, matrix remodelling and cell.cell adhesion
  - Cav1 and invadopodia formation
  - Cav1 and angiogenesis
- WHERE: stromal Cav1 and microenvironment
- Cav1 knockout mice models
- WHY: to hunt Cav1 in cancer and metastasis
  - Interaction with chemo agents and radiations
  - Exosomes: Cav1 as a new tumour marker?
- Concluding remarks

## Introduction

Caveolae (from the Latin word for ‘little cavities’) are 50–100 nm non-clathrin, flask-shaped invaginations of the plasma membrane that function as specialized membrane microdomains. Caveolae regulate signal transduction within the cell, as well as numerous other cellular processes including vesicular transport (transcytosis, endocytosis), cholesterol homeostasis, cell migration and the cell cycle [1]. Three small coat proteins have been identified in caveolae. Specifically, caveolin-1 (Cav1) and caveolin-2 (Cav2) are widely coexpressed in fully differentiated mesenchymal and endothelial normal tissues as well as in many solid tumours, whereas caveolin-3 (Cav3) is primarily expressed in muscle cells [2–4].

Caveolae represent one of the multiple raft endocytic pathways, not a major route of endocytosis, and act as an alternative to clathrin-coated pits. They contain several signalling molecules, such as G-proteins, non-receptor tyrosine kinases and endothelial nitric oxide synthase (eNOS) and function as organizing centres that concentrate key signalling transducers. Beside, caveolae seem to be directly involved in the cell response to mechanical stress: specifically, it has been reported that primary cell response to an acute mechanical stress occurs through the rapid flattening of caveolae into the plasma membrane [5]. It has been proposed that the complex relationship that links caveolae, caveolin and transmembrane signalling can be explained by the ‘caveolae signalling hypothesis’, in which the compartmentalization of signalling molecules within caveolae could allow for the efficient and rapid coupling of activated receptors to more than one effector system [6].

Recently, polymerase I and transcript release factor (PFTR; also known as Cav-p60 or Cavin), a soluble cytosolic protein, has been found to be required for caveolae formation [7]. PFTR-cavin is recruited to plasma domains containing phosphatidylserine (PS), cholesterol and oligomerized caveolins [7]. By binding these domains PFTR-cavin stabilizes the membrane curvature to produce the classic flask shape of caveolae. It indeed associates with mature caveolae at the plasma membrane but not with non-caveolar caveolin (in the Golgi complex or in mutant forms of caveolin) [7]. Interestingly, knockdown of PFTR-cavin reduces caveolae density and PFTR-cavin loss releases caveolar components, including caveolins, into the plasma membrane. Related proteins such as serum-deprivation protein response (SDPR)-cavin-2 and sdr-related gene product that binds to c-kinase (SRBC)-cavin-3 have also been reported to regulate membrane tubulation and caveola endocytosis: moreover, membrane remodelling and interdependent polarization of Cav1 and PTRF/cavin-1 in migrating cells has been recently shown to regulate cell migration [8–10].

Cav1 is an integral membrane 178-amino acid protein of 21–22 kD that was first identified in 1953. In 1992 Rothberg *et al*. named the protein caveolin and identified it as a unique array of filaments or strands that form striated coatings decorating the cytoplasmic surfaces of caveolae [11]. The Cav1 human gene is located on chromosome 7 in region q31.1 at the D7S522 locus [12]. The protein is synthesized in the endoplasmic reticulum (ER) and assembled along the membranes of the secretory pathway into large caveolar domains that subsequently travel towards the plasma membrane: recycling pathway for caveolae and caveolin is not yet clear. *In vivo*, two isoforms of Cav1 are known to exist: α-caveolin that contains the residues from 1 to 178 and β-caveolin that contains the residues from 32 to 178. Cav1 exerts its function by directly interacting with partner proteins through a defined, highly conserved region (aa 82-101) called the ‘caveolin-scaffolding domain’ (CSD) [3]. In this view, Cav1 could play a role of intracellular signalling modulator. Isoform α of the protein can be phosphorylated by Src on its tyrosine residue 14 [13, 14]. It is degraded through an ubiquitination process, and ‘caveosomes’ are late endosomal compartments that are modified by the accumulation of overexpressed Cav1 and awaiting degradation [15]. Recently, phospho-Cav1 has been reported to be a mechanotransducer that acts *via* PKC to relieve Egr1 transcriptional inhibition of Cav1 and cavin-1, thus defining a novel feedback regulatory loop to caveolae biogenesis [16].

The ability of Cav1 to modulate intracellular signalling has important implications in numerous human biological and pathological conditions, including tumourigenesis. In fact, during the past 20 years, several studies have carefully investigated the role of Cav1 in cancer initiation and progression, proving that this multifunctional protein regulates many cancer-associated processes, such as cell transformation, tumour growth, cell migration, invasion, multidrug resistance and angiogenesis. Despite this extensive body of work, our knowledge regarding the relationship between Cav1 function and tumourigenesis remains incomplete. The role of Cav1 in tumour onset and progression has been previously covered by several reports. The present review aims to highlight and discuss the most recent data which unveil the varied roles played by Cav1 during tumourigenesis, as growing evidence suggests that it may act both as inhibitor and promoter of growth signalling. Due to its versatile functions, the protein is now being exploited as a promising target for both cancer therapeutic and diagnostic approaches.

## WHEN: Cav1 expression and disease stage

In early studies, Cav1 expression was reported to be down-regulated in a wide range of human tumours and cell lines, which hinted at its tumour suppressor abilities [17–19]. In those settings Cav1 appears to be downregulated and seems to play a negative role in cancer transformation (Table 1). Many oncogenes, such as SRC, RAS, BCR-ABL, transcriptionally down-regulate Cav1 expression. Cerezo *et al*. have recently shown that Cav1-deficient fibroblasts show a faster escape from quiescence and progression through the cell cycle [20]. This effect is consistent with the potential role of Cav1 as a tumour suppressor. Cav1-negative cells features tumourigenic properties, such as the anchorage independent growth capacity, suggesting that loss of Cav1 regulation is an important step in the acquisition of the transformed phenotype.

**Table 1:** Caveolin-1 expression in primary carcinoma and in metastatic disease and correlation with *in vivo* and *in vitro* effects

	Clinicopathological correlations		
		
	*In vitro* effects	*In vivo* effects	References
*Cav1 in primary cancer*			
Cav1 tumour suppression			
Sarcoma bone and soft tissues	Increase anchorage independence, invasion and migration	Increase in the metastatic potential	[17]
GIST		Reduced expression (IHC), but no correlation with expression status and cell mitosis and tumour grade	[18]
Ovarian cancer	Suppression of tumour cell survival		[19]
Cav1 tumour promotion			
Prostate cancer		Gleason score, positive margins, aggressive cancer recurrence, lymph nodes involvement	[28, 35, 36]
RCC		Poor disease-free survival; higher mRNA Cav1 increase tumour stage; tumour size, TNM stage and grade	[29, 40]
Bladder carcinoma		Aggressiveness, tumour grade and stage	[30]
Brain tumour		*Oligodendroglioma*: Shorter survival *Ependymomas*: Unfavourable patient outcome	[31–33]
Breast carcinoma		Shorter disease-free and overall survival	[27]
NSCLC		Advanced pathological TNM stage and shorter survival	[37, 43, 45]
*Cav1 up-regulation associated with metastatic disease*			
RCC	Correlation with microvessel density, metastasis and poor prognosis		[40]
ESCC		Lymph node metastasis and worse prognosis	[41]
NSCLC	Induction of filopodia formation	Lymph node metastasis	[42, 43]
SCLC		Increase in the metastatic potential	[44]
Hepatocellular carcinoma	Promotion of cell proliferation, migration and invasion	Increase in the metastatic potential	[48, 49]
Malignant melanoma	Increase anchorage independence, invasion and migration		[38]
Ewing's sarcoma	Increased migration and invasion of the extracellular matrix	Increase in the metastatic potential	[47]

Cav1: caveolin-1; GIST: gastrointestinal stromal tumour; IHC: immunohistochemistry; RCC: renal cell carcinoma; ESCC: oesophageal squamous cell carcinoma; NSCLC: non-small cells lung carcinoma; SCLC: small cells lung carcinoma.

Supporting its role as a tumour suppressor gene, a sporadic dominant negative Cav1 mutation has been described in the literature. Hayashi *et al*. first reported a somatic punctiform Cav1 mutation at codon 132 (the P132L change that converts proline-132 into leucine) in 16% of the cases of primary human breast cancers that were examined [21, 22]. In contrast, *in vivo* experiments showed that Met-1 cells (a mouse luminal mammary epithelial cell line) harbouring *Cav1-1*_P132L_ formed tumours that were larger than Met-1 cells with wild-type Cav1 and had a greater metastatic potential [23]. Bonuccelli *et al*. has suggested that in primary tumour formation, *Cav*1*-1*_P132L_ acts as a loss-of-function mutation, whereas in the metastatic process, it behaves as a gain-of-function mutation promoting cell migration and invasion [24]. In fact, several signalling pathways (*e.g*. EGF, HGF and TGF-β) that are implicated in cell migration, invasion and metastasis appear to be up-regulated by *Cav1-1*_*P132L*_. Notably, structural analysis of other point mutations at position 132 of Cav1 (P132A, P132I, P132V, P132G, P132W, P132F) indicates that proline 132 is critical in supporting proper Cav1 behaviour [25]. However, it should also be noted that the existence of the P132L mutation has recently been questioned [26].

**Fig. 1:**
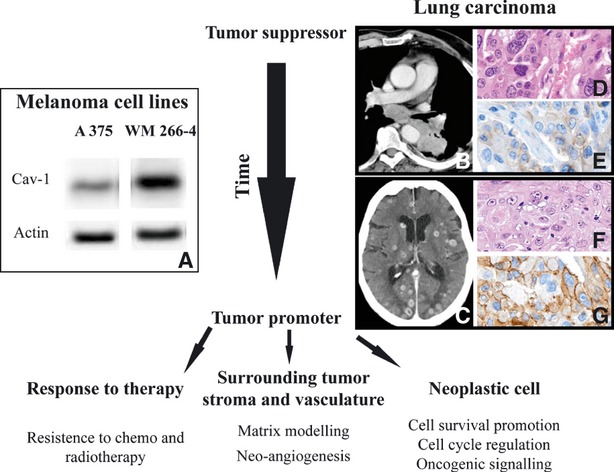
Effects of caveolin-1 activation that promote cancer progression. The left side of the figure shows the Western blot analysis of caveolin-1 expression in a metastatic (WM 266-4) compared with a non-metastatic melanoma cell line (A375; **A**). On the right of the figure, one case of a primary poorly differentiated lung carcinoma (**B**: TC scan; **D**: E&E, 40×; **E**: Cav1, 40×) and its corresponding brain metastasis (**C**: TC scan; **F**: E&E, 40×; **G**: Cav1, 40×) are reported. The primary lung lesion showed a focal and faint cytoplasmic and membrane Cav1 staining (D and E), whereas in the brain metastasis, (F and G) Cav1 expression was diffuse and intense.

Although Cav1 is likely to behave as a tumour suppressor protein in early phases of tumour onset, growing evidence suggests that more advanced or metastatic tumours have Cav1 activation (Table 1). In fact, Cav1 is commonly up-regulated in several advanced epithelial tumours including prostate, kidney, breast and bladder carcinomas and brain tumours [27–37]. In melanoma cell lines, Felicetti *et al*. have recently highlighted the tumour-promoting functional role of Cav1, demonstrating that high expression of phospho-Cav1 positively correlated with increased anchorage independence, invasion and migration [38]. Supporting these data, unpublished data from our group demonstrate increased Cav1 expression in metastatic compared with non-metastatic melanoma cell lines (Fig. 1). In addition, Sunaga *et al*. demonstrated that Cav1 expression was down-regulated in 95% of small cell lung cancer cell lines, which displayed Cav1 gene promoter hypermethylation. In contrast, Cav1 expression was maintained in 76% of non-small cell lung cancer (NSCLC) cell lines [39].

Up-regulation of Cav1 in some primary tumour cells has been reported to be associated with metastatic disease progression [40–48]. Sáinz-Jaspeado *et al*. proposed that Cav1 overexpression contributes to Ewing's sarcoma tumour cell migration, invasion of the extracellular matrix and metastasis [47]. In hepatocellular carcinoma, Cav1 up-regulation seems to promote cell proliferation, migration and invasion *in vitro* and metastatic potential *in vivo*.[48]. Similarly, elevated levels of Cav1 increased cell motility and invasion in the human hepatocellular carcinoma cell lines (HepG2 and Huh7), and caveolae disruption reduced SNU-449 and SNU-47 cell motility and invasion [49]. In lung adenocarcinoma cell lines, the enhanced metastatic potential associated with Cav1 up-regulation depends on the induction of filopodia formation [42].

The contribution of Cav1 to cancer progression appears to be complex and remains controversial. In an attempt to resolve the conflicts between the biological and functional data, it has been suggested that in the early stages of cancer progression Cav1 restrains tumour growth, whereas in the advanced stages of disease, Cav1 has a pro-survival and tumour promoting role [50]. According to this view, Cav1 could play a role as either a tumour suppressor gene or an oncogene, and the switch is likely governed by tumour stage and tumour temporal context [51].

## HOW: Cav1 may promote tumour spreading

Cancer is a multistep genetic and epigenetic disease that results from the progressive accumulation of mutations which either inactivate tumour suppressor genes or activate dominant proto-oncogenes [52]. These aberrant events free cells from proliferative control and allow primary tumour formation. The initial tumour growth is followed by metastatic spread, and ultimately, metastases that are resistant to conventional therapies represent the major cause of death from cancer.

As discussed above, the role played by Cav1 in cancer and progression has not been fully clarified. Although lacking a fundamental mechanistic understanding to explain a fully Cav1-induced malignant phenotype, it is well documented that Cav1 cooperates with other proteins to promote cancer dissemination by affecting critical functions of cellular maintenance and homeostasis. During the early stages of tumour progression, Cav1 negatively controls cell-cycle progression and restrains cell proliferation, whereas growing evidence suggests that Cav1 may induce more advanced cancer phases [3]. Cav1 interacts with a series of molecules/receptors which do not specifically function in caveolae, but which regulate initial steps of cells transformation as well as their metastatic potential [3, 53]. It should be noted that not all the data reported below have been independently repeated in multiple model systems, therefore not all have the same significance.

### Cav1 and cell-cycle modulation

Cyclin D1 belongs to a family of proteins that promotes cancer cell progression through the G1-S phase of the cell cycle by binding the cyclin-dependent kinase CDK4. This interaction ultimately induces phosphorylation of the retinoblastoma protein pRb, with release of the E2F transcription factor allowing cells to progress in the cell cycle [54]. This phenomenon been identified in several epithelial tumours, including breast, colon, ovarian, prostate and lung tumours. Importantly for human metastatic lung adenocarcinoma and small cell lung cancer (SCLC), it has been reported that cyclin D1 expression is regulated by Cav1 expression and aberrant activation of the signal transducer and activator of transcription-3 (STAT3) [55]. Cav1 silencing induces the stable arrest of proliferation in metastatic lung cancer cell lines through a significant decrease in both cyclin D1 expression and phosphorylated AKT expression, as well as a decrease in its downstream transducers phosphorylated ERK and STAT3 [49]. STAT3 is an oncoprotein that is frequently hyper-activated by phosphorylation of a conserved tyrosine residue in response to extracellular signalling molecules such as cytokines and growth factors [56]. Conversely, it has been demonstrated that STAT3 can regulate the expression and function of Cav1 by directly binding the Cav1 promoter and inhibiting its transcription [57]. These findings reveal a reciprocal network between Cav1 and STAT3 that regulates distant cancer metastasis. Forkhead box M1 (FoxM1) is a transcription factor that plays a role as regulator of cell-cycle phases (G1/S and G2/M), angiogenesis, invasion and metastasis [58]. Recently, a direct correlation between Cav1 and FoxM1 expression has been reported in pancreatic cancer cells [59]. High Cav1 expression correlated with poor tumour differentiation, advanced tumour stage and distant metastasis. In addition, Cav1 and FoxM1 expression directly correlated, and FoxM1 positively transactivated Cav1 activity by binding to the promoter region.

### Cav1 and growth factors signalling

Another mechanism by which Cav1 may affect cell fate is by its ability to interact with growth factors and cytokines. With respect to growth factors, many previous studies have linked Cav1 to the epidermal growth factor (EGF) and EGF receptor (EGFR) pair. Although Cav1 seems to down- regulate signal transduction, its phosphorylated form has been associated with EGF-induced cell migration and anoikis [3, 60]. On the other hand, EGF induces tyrosine phosphorylation of Cav1, which, in turn, is essential for EGFR transactivation and AKT signalling [61]. Cav1 can also interact with the insulin growth factor receptor (IGFR) by its ability to bind cholesterol, and receptor regulation has been linked to the insulin-induced tyrosine phosphorylation of Cav1. Cav1 phosphorylation leads to the caveolar recruitment of several SH2 residue containing proteins, such as Grb7, that are responsible for IGFR activation [62, 63].

### Cav1 and Rho-GTPases

Rho-GTPases are members of the RAS superfamily of small guanosine triphosphatases (GTP-ases) that participate in the regulation of cell movement and migration by controlling actin-myosin filament assembly to form stress fibres and maintain focal adhesion [64]. Rho induces the binding of Cav1 to actin cross-linking protein filamin A, which produces an alignment between structures that are positive for Cav1/filamin and stress fibres. This suggests that Cav1 plays a role at the interface of the cytoskeleton [65]. Cav1 could regulate cell polarization and directional migration through a process involving Src and Rho-GTPases [66].

### Cav1 and integrins

Integrins physiologically regulate cell adhesion by binding to extracellular proteins. Cav1 is a protein that physically represents a link and functions as an adaptor between integrins and tyrosine kinase signalling [67, 68]. Tyrosine phosphorylated Cav1 (pY14Cav1), which is virtually extracaveolar, is associated with Rho activation and Src-dependent regulation of tumour cell motility, stabilization of focal adhesion kinases (FAK) within focal adhesions (FAs) and enhancement of tumour cell migration and invasion [69]. Recently, Arpaia *et al*. demonstrated that the interaction between Cav1 and Rho-GTPases promotes metastasis by stimulating the expression of α5-integrin [70]. Beside, Cav1 phosphorylated on tyrosine 14 contributes to adhesion maturation and tumour cell migration through a crosstalk with galectin-3 [71]. Indeed galectin-3 binding to Mgat5-modified N-glycans functions together with pY14Cav1 to stabilize FAK within FAs, and thereby promotes FA disassembly and turnover.

Recently, it has been demonstrated in glioblastoma that Cav1 controls α5β1 integrin expression by the TGFb/TGFbRI/Smad2 pathway [72]. Cav1 acts as a negative regulator of α5β1 integrin expression in glioma cells and specifically, loss of Cav1 confers a more aggressive phenotype [73]. In addition, Cav1 depletion can block the association between Src kinases and ß1 integrin, resulting in both the loss of focal adhesion domains that mediate the interaction between the ECM and cytoskeletal proteins and increased tumour migration [74]. Recently, Cav1 has been reported to reduce metastatic potential in melanoma cells through the suppression of the Integrin/Src/FAK signalling pathway [75].

### Cav1, matrix remodelling and cell–cell adhesion

The metastatic potential of malignant cells relies on their invasion and migration abilities in response to changes in the extracellular matrix surrounding the tumour. Physiologically, Cav1 controls cell motility by interfering with the cytoskeleton and modulating cell interaction with the extracellular matrix [76]. In fact, Cav1 regulates matrix degradation and interacts with several molecules involved in cell motility and dynamics [3].

Caveolin-1 participates in the remodelling of the extracellular matrix (ECM) by promoting interactions with matrix metalloproteinases (MMPs). MMPs are a family of zinc-containing proteolytic enzymes that play a critical role in tumour invasion and metastasis by favouring the degradation of different extracellular matrix proteins [77]. Among all MMPs, MMP9 and MMP2 (gelatinase A and B) are reported to be frequently overexpressed in cancer progression and positively associated with metastases [78]. Although Cav1 has been shown to decrease MMP2 and MMP9 activity, Cav1 can be up-regulated during Epithelial-Mesenchymal Transition (EMT) by acting on other members of the MMP family [79–81]. For example, Cav1 overexpression, in association with the secretion of MMP3 and MMP11, enhances nasopharyngeal carcinoma cell migration [82]. Moreover, Cav1 may affect cancer cell adhesion by modulating E-cadherin (E-cad), which is a major component of adherens junctions [83]. Cadherin-mediated adhesion plays a crucial role in maintaining cell–cell contacts and reducing metastatic disease. The binding of β-catenin to membrane E-cad is a prerequisite for cell adhesion because the cytoplasmic domain of E-cad binds β-catenin, and the resulting complex is linked through α-catenin to the cytoskeleton [84, 85]. Loss of E-cad is a prerequisite for migratory activity and development of an invasive metastatic phenotype in cancer [86]. Cav1 and E-cad co-localize and interact at the cell surface. The connection between the two molecules may differ according to the different cell types where they are expressed. Some reports documented a Cav1 mediated increase in cell–cell adhesion, in association with the stabilization of adherens junctions and E-cad expression [87, 88]. Interestingly, in lymph node metastases from head and neck squamous cell carcinomas (HNSCCs), Cav1 overexpression has been associated with the abnormal expression of at least one member of the E-cad/α-β catenins complex, as well as multiple ErbB receptors [89].

### Cav1 and invadopodia formation

Invadopodia are ventral membrane protrusions that can degrade the extracellular matrix, mainly through membrane type 1 matrix metalloproteinase (MT1-MMP) activity [90]. Their role is important during metastatic and invasive processes. Cav1 seems to be able to regulate invadopodia-mediated extracellular matrix degradation [91]. In fact, Cav1 accumulates at invadopodia and both co-localize and co-traffic with MT1-MMP. Cells with reduced Cav1 expression showed a significant decrease in invadopodia formation and gelatin degradation activity [83, 84].

### Cav1 and angiogenesis

To obtain the blood supply necessary for their growth and overcome ensuing local hypoxia and metabolic needs, tumour cells can tilt the balance towards stimulatory angiogenic factors and drive vascular growth. Neovasculature generated by tumour cells is typically tortuous and permeable, features that favour intravasation, a very early event in the metastatic process that is represented by entrance of tumour cells into the bloodstream. Neoangiogenesis is a multifaceted process governed by several factors. Among them, vascular endothelial growth factor (VEGF) regulates angiogenesis by the complex interaction of cellular-signalling pathways. VEGF secreted by tumour cells activates endothelial cells (ECs) in a paracrine manner and sets in motion all of the functions that are required for angiogenesis, including matrix degradation, migration, proliferation and the sprouting of new vessels from the existing ones. VEGF-induced angiogenesis and hyperpermeability require nitric oxide (NO) that is produced by the activity of nitric oxide synthase (eNOS), a NOS isoform known to be concentrated in caveolae in endothelial cells [92]. Cav1 is able to interact with eNOS by inducing inhibition of NO synthesis. Moreover, reactive oxygen species (ROS) are now widely recognized to contribute to both cell homeostasis and cancer, and NO contributes to oxidative stress [93]. eNOS is the main source of vascular NO, and aberrant regulation of eNOS activity is linked to a range of vascular diseases and to the pro-angiogenic activity that characterizes many solid tumours. Cav1 acts as an inhibitory clamp that maintains eNOS in an inactive state until haemodynamic forces that are transmitted into the caveolae, uncouple eNOS from caveolin for activation. Thus, the inhibitory effect of Cav1 on eNOS may have an impact on vascular permeability and angiogenesis.

The relationship between the tumour angiogenesis promoted by Cav1 and the enhanced metastatic potential of a neoplastic clone has yet to be elucidated. Cav1-deficient mice show a constitutive eNOS activation which results in increased microvascular permeability: down-regulation of Cav1 is predicted to promote uncontrolled eNOS activity, thereby facilitating tumour onset and stimulating tumour vasculature by inducing the expression of angiogenic growth factors that regulate endothelial cell growth and tubule formation [94, 95]. In multiple myeloma, VEGF secreted by neoplastic cells is able to induce a paracrine Src-mediated Cav1 phosphorylation [96]. In hepatocarcinoma (HCC), overexpression of Cav1 has been associated with the overexpression of VEGF and the increase in microvessel density in unpaired arteries. Thus, angiogenesis may be affected by Cav1, and through this mechanism it facilitates metastatic dissemination in HCC [97].

## WHERE: stromal Cav1 and microenvironment

Tumours are made up of a mixed population of cells that includes normal structures adjacent to transformed clones, and both interact with the microenvironment. Mechanical forces driving these interactions cooperate in tumour invasion and metastasis, and cancer-associated fibroblasts can biomechanically remodel the extracellular matrix surrounding the tumour. Recently, several authors have focused their attention on Cav1 expression in the tumour stromal cells (stromal Cav1) rather than Cav1 cellular expression, as evidence suggests that there may be an important role for stromal Cav1 in promoting tumour progression and metastasis. Goetz *et al*. reported that stromal Cav1 is a regulator of matrix-dependent cell behaviour, highlighting its ability to facilitate invasion and metastatic potential through a regulation of Rho activity and p190 localization and phosphorylation [98]. Consistent with these data, the expression of Cav1 in carcinoma-associated fibroblasts facilitates *in vitro* directional migration and invasiveness. However, this finding appears to contrast previous reports that propose that the absence of stromal Cav1 is a negative prognostic factor in cancer [99]. The absence of stromal Cav1 has been reported to predict early tumour recurrence and lymph node metastases and to be a predictor of poor clinical outcome in breast cancer [100]. In addition, loss of stromal Cav1 was associated with the disease progression of invasive breast cancer in ductal carcinoma *in situ* [101]. Similarly, high stromal Cav1 levels have been demonstrated to correlate with reduced metastasis and improved survival [102]. Recently, high Cav1 cell expression, in association with an absence of Cav1 stromal expression, has been reported to be closely associated with poor outcome in a subset of breast cancer patients [103]. A deficiency of stromal Cav1 in human prostate cancer has been identified as a biomarker for tumour progression to metastatic disease as well [104]. Reduced stromal Cav1 expression has also been reported to label a subgroup of patients that have an unfavourable survival prognosis and aggressive malignant melanoma metastases [105]. Sotgia *et al*. have recently demonstrated that Cav1−/− fibroblasts secrete high levels of pro-angiogenic or pro-tumourigenic factors, such as VEGF, platelet-derived growth factor, MET, interleukins and chemokines, which have been shown to support metastatic disease [106]. This mechanism could partially explain why the loss of stromal Cav1 in cancer-associated fibroblasts correlates with metastatic dissemination. In addition, a loss of stromal Cav1 has been reported to lead to the overexpression of plasminogen inhibitor type 1 and 2 (PAI-1 and PAI-2), likely as a consequence of increased oxidative stress. Cancer cells activate transcription factors in adjacent stroma *via* oxidative stress (Warburg effect), which represents a crucial step for cancer progression and dissemination. Stromal cells lacking Cav1 undergo aerobic glycolysis and secrete energy-rich metabolites that directly feed cancer cells and fuel mitochondrial respiration of adjacent cells through the phenomenon called the ‘reverse Warburg effect’ [107].

To date, the discordant role of stromal Cav1 on tumour progression and metastasis appears to be poorly understood although several mechanisms have been proposed by different authors to explain its critical function as a regulator of tumour-surrounding tissue remodelling and desmoplastic processes [98, 108].

## Cav1 knockout mice models

Truly caveolae-deficient mice have been generated and contributed to assess Cav1 functions *in vivo* in the context of a whole animal. Moreover, a knockout mouse phenotype is often different from a drug inhibitor phenotype as the latter is usually partial. Notably, loss of a major cellular organelle imparts no developmental abnormality or lethality.

Interestingly, Cav1 (−/−) null mice completely lack caveolae, while ectopic expression of Cav1 is sufficient to induce caveolae in cells that were previously lacking these organelles [109]. Knockout animals displayed severe dysfunction of the vascular system. Surprisingly, the deletion of these organelles was not lethal, a pronounced thickening of lung alveolar septa caused by an uncontrolled fibrosis. Cav1^−/−^ mice demonstrate organ-specific growth-related disorders in stromal cells that normally have high levels of Cav1 expression [110]. In many of these organs, epithelial cell growth/differentiation abnormalities were also observed, yet in most of these sites the epithelial cells normally express low to non-detectable levels of Cav1. This observation suggests that loss of Cav1 function in stromal cells of various organs directly leads to a disorganized stromal compartment that, in turn, indirectly promotes abnormal growth and differentiation of adjacent epithelium.

Caveolin-1-deficient mice feature some interesting pathophysiological changes, with clearly abnormal lungs featuring airway hyper-reactivity and a hypotonic vasculature [111]. Park *et al*. reported that double-knockout Cav1 and Cav3 mice are viable and fertile, although they lack morphologically identifiable caveolae in endothelia, adipocytes, smooth muscle cells, skeletal muscle fibres and cardiac myocytes [112]. Authors also show that those mice are deficient in all three caveolin gene products, as Cav2 is unstable in the absence of Cav1. Interestingly, dual ablation of both Cav1 and Cav3 genes in mice leads to a pleiotropic defect in caveolae formation and severe cardiomyopathy with interstitial/perivascular fibrosis. Thus, Cav1^−/−^ mice are markedly abnormal, although Cav1 is not expressed in cardiac myocytes. However, Cav1 is abundantly expressed in the non-myocytic cells of the heart, such as cardiac fibroblasts and endothelia. In addition, endothelial and iNOS levels are dramatically up-regulated [113]. Besides Cav1^−/−^ mice may have problems with lipid metabolism and/or adipocyte functioning [114]. Although serum insulin, glucose and cholesterol levels are entirely normal, Cav1 null mice have severely elevated triglyceride and free fatty acid levels, especially in the post-prandial state. This is consistent with previously shown functions of Cav1 in lipid metabolism and homeostasis [115]. In addition, although Cav1 plays a role in several oncogenic pathways, Cav1 null mice do not present a higher incidence of carcinomas. Coherently,—as Cav1 does not directly play as oncogene or full tumour suppressor molecule—they do not show signs of hyperproliferation. Very recently, it has been shown that genetic ablation of Cav1 differentially affects melanoma tumour growth and metastasis in mice, as Cav1-deficient dermal fibroblasts are able to promote the growth of melanoma cells *via* enhanced paracrine cytokine signalling [116]. In contrast the ability of melanoma cells to form lung metastases seems to be significantly reduced in Cav1KO mice: this behaviour has been linked to the inability of melanoma cells to adhere to and to transmigrate through a monolayer of endothelial cells lacking Cav1.

## WHY: to hunt Cav1 in cancer and metastasis

As discussed above, Cav1 plays a key role in cell signalling and in tumour progression towards the metastatic stage. Several strategies are now under investigation to target Cav1 for the prevention of tumour progression and dissemination: all of them attempt to exploit the unique properties of Cav1 in molecular trafficking, and include targeting Cav1 using antisense and siRNA approaches, re-expressing ectopic caveolin, introducing a CSD and lowering cholesterol with statins or other drugs. Targeting vascular endothelium is a relatively novel approach to tumour therapy, and the selective endocytosis mediated by Cav1 represents one of the most promising current strategies to achieve localized gene expression and direct damage of endothelial cells. Indeed, caveolae provide a unique vascular pathway for both selective uptake of molecules and their delivery to specific cells and tissues. Targeted gene delivery of Cav1 has been undertaken using a different approach to alter Cav1 expression in vascular endothelial cells. A recently developed cell-permeable peptide, derived from the amino acids of the CSD of Cav1 and termed cavtratin, has been shown to reduce microvascular hyperpermeability and to delay tumour progression in mice by inhibiting eNOS [117]. Moreover, cavtratin inhibits the phosphorylation of the NGF receptor TrkA as well as the activity of downstream components of the NGF signalling pathway in oligodendrogliomas [118]. The cell-permeable peptide is likely not a true drug candidate but a tool. In this perspective, the CSD of caveolin may also be considered as a novel therapeutic target. On the other hand, caveolae can be utilized as a useful transport pathway to improve tissue-directed drug and gene delivery. This is an interesting approach, particularly if one considers that caveolins affect sensitivity to chemotherapy. In addition, caveolae provide a novel vehicle to deliver imaging agents and nanoparticles to specific tissues *in vivo*. Higher Cav1 expression in advanced tumours is exploited by the recently developed nanoparticle albumin-bound (nab) technology, which promises to have broad utility in cancer therapy and proposes a mechanism to deliver nab-driven chemotherapy that exploits the cargo properties of Cav1.

The nano-shuttle system is believed to activate an albumin-specific (Gp60) receptor-mediated transcytosis pathway in the neoplastic cell by using Cav1-activated caveolar transport [119]. In addition to caveolae and caveolins, targeted agents could be used as promising tools for imaging to identify the primary tumour and metastatic lesions and to localize metastatic lesions and vascular patterns of neoplastic embolization.

### Interaction with chemo agents and radiations

The Cav1 role in radio- and chemoresistance of tumour cells also provides rationale for targeting Cav1 in cancer. The phenomenon of pharmaco- and radioresistance is still a major open issue in cancer treatment that has prompted studies to clarify the mechanisms of drug action and anti-neoplastic resistance (Fig. 1). Cellular levels of Cav1 have been shown to be increased in multidrug resistant cancer cells [120]. Cav1 knockdown sensitizes human renal carcinoma cells to doxorubicin-induced apoptosis and reduces lung metastasis in a mouse model [121]. Exposure to taxol, a molecule that stabilizes microtubules, may affect the cycling of Cav1 between the plasma membrane and the Golgi apparatus [122]. Taxol induces Cav1 up-regulation in A549 cells in an independent manner from the expression of P-glycoprotein [123]. Cav1 up-regulation following exposure to taxol may represent a way for the cell to compensate for the effect of the drug on the microtubule cytoskeleton. A connection between Cav1 and the DNA repair process has been demonstrated, given that silencing of Cav1 increased the levels of residual DNA double-strand breaks in irradiated 3D cell cultures, and Cav1 knockdown sensitized pancreatic tumour cell lines grown in 3D lrECM to X-rays [124]. Cav1 expression has also been shown to be significantly associated with poor prognosis and drug resistance in advanced non-small cell lung cancer patients after treatment with gemcitabine [125]. Interestingly, Dittmann *et al*. showed that Src kinase activation following irradiation triggers Cav1-dependent EGFR internalization into caveolae [126]. Therefore, EGFR is able to reach the nucleus and bind to DNA-dependent protein kinase (PK), which is an essential mechanism of non-homologous end-joining DNA repair. Treatment with cetuximab, which binds to the extracellular domain of EGFR, results in receptor internalization and formation of an intracellular complex of EGFR, Cav1 and the monoclonal antibody. Radiation-induced activation and nuclear translocation of EGFR are mediated through Src kinase activity in a Cav1-dependent process. A different mechanism through which Cav1 is involved in the response to ionizing radiation is related to the interaction of Cav1 with focal adhesion proteins. This process has demonstrated that Cav1-silenced pancreatic cancer cells seem to be sensitized to ionizing radiation, and this suggests that Cav1 acts as pro-survival factor in the cellular response to ionizing radiation [127]. In addition, enhanced radiosensitivity of Cav1-deficient mice has been associated with increased apoptosis and abnormal proliferation of intestinal crypt stem cells of the small intestine, resulting in increased susceptibility to γ-radiation [128].

The recent discovery of Cav1 as a metastasis promoter has challenged its role as biomarker of tumour progression. As discussed above, the up-regulation of intracellular Cav1 is associated with the activation of several pro-survival and pro-invasive pathways. Nevertheless, there is a growing interest in secreted Cav1 as a biologically active molecule that promotes cell survival and angiogenesis within the tumour microenvironment. Secreted Cav1 can be reproducibly detected in peripheral blood by using specific assays. The ultimate goal of such an approach is to allow patient stratification into metastasis-specific risk categories based on Cav1 levels.

### Exosomes: Cav1 as a new tumour marker?

Exosomes are 30–100 nm small membrane vesicles containing cytosolic and membrane proteins that are released through an exocytosis pathway by most cell types [129]. Their release appears to be finely modulated under both physiological and pathological conditions. Exosome release has been found to be dramatically increased in cancer [130]. Thus, exosomes have been proposed to play an important pro-tumourigenic role, facilitating both tumour progression and metastasis by modifying the host microenvironment. In recent years, exosome release by neoplastic cells in biological body fluids, such as blood and urine, has been extensively investigated. Cav1 has been reported to be involved in prostasome (vesicular organelles secreted by prostate epithelial cells) secretion in the human prostate cancer cell line PC-3 [131]. More recently, Logozzi *et al*. have demonstrated a strong expression of Cav1 in exosomes secreted by human melanoma cells *in vitro* and in those obtained from plasma of SCID mice engrafted with melanoma tumours: on the contrary, it is undetectable in cellular extracts and in exosomes from normal human cells such as primary monocyte-derived macrophages [132]. Authors observed that a significant increase in exosomes expressing tumour markers such as Cav1 can be observed in the plasma of melanoma patients with respect to healthy individuals. As a consequence, Cav1 plasma levels could bear relevance as a possible prognostic marker in melanoma patients, and exosomes may represent a hallmark of more aggressive melanoma that identifies patients with poor outcome. Besides authors findings suggest that an exosome-specific ELISA assay may be used to detect and quantify circulating exosomes in melanoma patients [132]. However, further investigations are still necessary to understand the role of Cav1 in exosome biology.

## Concluding remarks

Caveolin-1, a principal structural component of caveolar membrane domains, contributes to cancer development, but its precise role in cancer regulation remains unclear. Growing evidence supports the hypothesis that Cav1 is switched on during neoplastic progression, and its overexpression characterizes the most advanced stages of several malignant diseases. Cav1 acts as cargo molecule and orchestrates the trafficking across the cell plasma membrane, which is required for metastasis. Importantly, Cav1 plays a dual regulatory role in controlling microvascular permeability: (i) as a structural protein that is required for caveolae formation and caveolar transcytosis and (ii) as a tonic inhibitor of eNOS activity to negatively regulate the paracellular pathway. Finally, Cav1 expression can mediate chemo- and radioresistance of tumour cells. Overall Cav1 is a promising target for selective cancer diagnosis and therapy as modulator of intracellular signalling. In particular, Cav1 targeting could promote the penetration of anticancer drugs, gene vectors or imaging probes through the endothelial cell barrier.
